# Epistemic Injustice in Incident Investigations: A Qualitative Study

**DOI:** 10.1007/s10728-022-00447-3

**Published:** 2022-05-31

**Authors:** Josje Kok, David de Kam, Ian Leistikow, Kor Grit, Roland Bal

**Affiliations:** 1 Erasmus School of Health Policy and Management, Erasmus University, P.O. Box 1738, 3000 DR Rotterdam, The Netherlands; 2 Antoni van Leeuwenhoek / The Netherlands Cancer Institute, Plesmanlaan 121, 1066 CX Amsterdam, The Netherlands; 3 Dutch Health and Youth Care Inspectorate (HYCI), P.O. Box 2518, 6401 DA Heerlen, The Netherlands

**Keywords:** Incident investigations/Root Cause Analysis, Epistemic injustice, Patient and family involvement, Professional involvement, Incident reporting systems

## Abstract

Serious incident investigations—often conducted by means of Root Cause Analysis methodologies—are increasingly seen as platforms to learn from multiple perspectives and experiences: professionals, patients and their families alike. Underlying this principle of inclusiveness is the idea that healthcare staff and service users hold unique and valuable knowledge that can inform learning, as well as the notion that learning is a social process that involves people actively reflecting on shared knowledge. Despite initiatives to facilitate inclusiveness, research shows that embracing and learning from diverse perspectives is difficult. Using the concept of ‘epistemic injustice’, pointing at practices of someone’s knowledge being unjustly disqualified or devalued, we analyze the way incident investigations are organized and executed with the aim to understand why it is difficult to embrace and learn from the multiple perspectives voiced in incident investigations. We draw from 73 semi-structured interviews with healthcare leaders, managers, healthcare professionals, incident investigators and inspectors, document analyses and ethnographic observations. Our analysis identified several structures in the incident investigation process, that can promote or hinder an actor’s epistemic contribution in the process of incident investigations. Rather than repeat calls to ‘involve more’ and ‘listen better’, we encourage policy makers to be mindful of and address the structures that can cause epistemic injustice. This can improve the outcome of incident investigations and can help to do justice to the lived experiences of the involved actors in the aftermath of a serious incident.

## Introduction

Learning from the (serious) incidents that occur in the complex realm of healthcare delivery has been a top priority in healthcare since the publication of the influential report *To Err is Human* [30]. Accordingly, over the years incident investigations have become a consistent—often legally mandated—component of national and local incident reporting systems around the world [32]. In the American, Australian, English and Dutch healthcare systems, serious incident investigations are routinely conducted using Root Cause Analysis (RCA) tools [26, 35]. RCA is the process of discovering the root and latent cause(s) of an incident. Different RCA tools can be used, such as the ‘5 Why’s approach’, a ‘Timeline’ or ‘Fishbone diagram’. Although these tools differ, they have in common that they are highly methodological approaches that consist of a structured retrospective analysis to find cause-effect evidence, focusing on ‘how’ and ‘why’ something has happened (not ‘who’ is responsible). Based on the root causes, improvement measures are formulated in an attempt to minimize the risk of reoccurrence [38].

RCA tools adopted by healthcare organizations stimulate incident investigators to collect input from all actors who are most familiar with, have knowledge about and/or were part of the incident [46]; professionals, patients and their families alike. Several ideals underpin the appeal for such broad inclusiveness. First, we live in an age of transparency, wherein healthcare organizations are publicly called upon to be open about medical error and other types of patient safety incidents [23, 45]. Including all actors in an investigation then, is a way of being accountable, doing the ‘right’ thing [31], being transparent and open. Secondly, there are the widely embraced ideals of patient-centered care and system-based learning, wherein all actors are seen as experts in their own right, who bring valuable knowledge that can inform learning from what has gone wrong and improve healthcare services more generally [3, 31]. Particularly patients and their families are increasingly recognized as invaluable informants when it comes to healthcare improvement. It has been noted they make important observations that are different from those of managers and healthcare staff [1, 3, 18, 29], fueling calls to take user experiences—their complaints and ideas for service improvements—seriously. Harvesting different experiences in incident investigations is also an imperative when learning is conceived of as a social and participative process that involves people actively reflecting on shared knowledge and practices [33]. Alongside this learning perspective, involving and hearing all knowledgeable actors is also presented as the right thing to do—for patients, their families and caregivers,—facilitating healing and closure [7, 10, 31, 47]. Importantly, within this latter ideal, allowing participants to provide testimony is not about who is ‘objective’, ‘right’ or more reliable, but about providing room for the perspectives of all those involved.

Even though the value of involving multiple actors in incident investigations is increasingly recognized—and publicly called for, in this age of transparency—it does not always happen [14, 20]. Moreover, where there is multi-voiced involvement to improve healthcare services and quality of care, research has shown that gathering, processing and assigning value to multiple perspectives is a challenging endeavor. A recent interview study (a qualitative study using interviews for data collection) revealed that healthcare staff often view patients as unreliable commentators on the quality of care given, as well as lacking insight on their own care and treatment priorities [1]. These opinions negatively influence the way patient narratives—specifically patient complaints—are assessed and used by medical professionals to reflect on services [1]. A study in English National Health Service (NHS) hospitals reported that dissent and conflict between different professional voices harvested in the investigation was frequently edited out of the final investigation report [38]. Similarly, a study in Dutch hospitals concluded that although patient and family members were routinely involved in incident investigations, their input was often downplayed and when the contributions of patients and families contradicted that of professionals, the professional perspective prevailed [31].

Muting or underrating voiced experiences, as demonstrated in the above-mentioned studies, potentially hampers social learning and improving health services. Moreover, not feeling heard or listened to in the aftermath of a (serious) incident can inflict intensified grievance [10, 28], inhibit reconciliation and cause patients and their families to go on a ‘legal crusade’[37]. To understand why it is difficult to assign value and learn from all perspectives, we (re)examined qualitative data collected in research projects on serious incident investigation practices in Dutch healthcare organizations. We studied these incident investigation practices to identify instances where professionals, patients and their families are prone to experience ‘epistemic injustice’.

Developed by Fricker, the concept of ‘epistemic injustice’ refers to a wrong done to someone specifically in their capacity as a knower [17]. Fricker identifies two types of epistemic injustice: testimonial and hermeneutical. First, testimonial injustice occurs when the speaker receives a credibility deficit owing to identity prejudice in the hearer. In other words, the speaker’s knowledge is disqualified not because of what the speaker says, but because of the hearer’s prejudice towards the speaker. An example could be if a doctor doesn’t take an adolescent-patient’s input seriously when discussing treatment options due to her young age. In this example, the patient suffers a credibility deficit, not because she has articulated her opinion unclearly, but because of the age-based stereotype attributed to her by the hearer (the doctor thinks adolescents are too young to know what is in their best interest). The second type, hermeneutical injustice, occurs when a lack of resources, usually conceptual resources, puts someone at an unfair disadvantage when it comes to making sense of, and sharing, their experiences [17, 24]. Fricker tells the story of a woman who suffered injustice as she battled depression succeeding the birth of her son. For years she blamed herself and was blamed by her husband, until she attended a meeting in the late 1960s where postpartum depression was discussed. Here the unveiled conceptual resource—the linguistic label ‘postpartum depression’—enabled the woman to understand her condition, previously ill-understood by herself as well as others. Should the woman have visited a therapist before the condition ‘postpartum depression’ was officially taken up in the Diagnostic and Statistical Manual of Mental Disorders, the therapist may have dismissed her claims. In this example then, the woman’s husband and therapist—the hearers—may not have been unwilling to believe the woman’s testimony, but they could simply not understand what she was saying as they may have lacked the same concept [17]. Certain groups of people, Fricker adds, are more prone to be hermeneutically disadvantaged—and suffer hermeneutical injustice—for social norms, cultural practices, structural social inequalities and institutional arrangements prevent them equal access to the resources to make sense of and articulate their experiences, and epistemic injustice tends to reproduce or even enlarge such inequalities as it prevents people to join in decision-making that also affects their position [2, 5, 17]. In a related vein, when someone is not granted access to participate in epistemic activities at all, this is called epistemic exclusion [5, 24]. Taken together, the notions of epistemic injustice and exclusion are insightful when studying quality and safety improvement efforts, as they allow us to recognize how potentially valid knowledge is undervalued and/or not shared [42].

The concept of epistemic injustice has been used in empirical studies in healthcare before. These studies mainly focus on care related encounters between health professionals and patients—in pregnancy and childbirth [16] or surrounding the contested medical status of chronic fatigue syndrome [4]. They show how power imbalances between both groups renders patients prone to suffer epistemic injustice in these encounters [5]. In addition to the social interactions in healthcare, epistemic injustice can also be triggered through the structures of contemporary healthcare practices, as organizations and procedures privilege certain styles of articulating testimonies, certain forms of evidence, and certain ways of presenting and sharing knowledge [5, 36]. When we recognize that the specific structure of incident investigations encourages a specific way of talking about, thinking about and doing safety [2], we expect that some actors invited to contribute to incident investigations are prone to suffer epistemic injustice when their testimonies do not suit this structure. Fueling this expectation are the earlier described studies demonstrating that incident investigators occasionally neglect or silence different interpretations offered during the investigation process [31, 38]. Moreover, other scholars have reported that professionals and patients sometimes feel frustrated and unjustly treated because their accounts are not (fully) sought out [37], are misrepresented or missing entirely in the final reconstruction, drawn up by incident investigators [7, 25, 26, 28, 38, 41]. These insights at the very least show that the concept of epistemic injustice can draw relevant insights for analyzing incident investigations.

Over the years, patient safety literature has often reiterated calls to value the input of patients and families, to take them seriously [28, 41], and to do justice to the experiences of professionals [7]. These repeated calls, however, do not adequately take into account the structural characteristics of healthcare that might thwart such efforts, nor do they look at the effects of specific instrumentations like the RCA and how these affect the position of concerned actors. In what follows we thus use the concept of epistemic injustice to help us understand and identify the specific instances in incident investigation routines where participants are prone to experience an unjust disqualification of their testimony. It is important to stress that, as Moes recently noted, while epistemic injustice is a normative concept, this is not a normative analysis [36]. It is *not* our intent to make normative judgments or injustice claims on behalf of patients, families or professionals regarding specific knowledge claims; rather this is an empirical analysis in which we seek to identify the structural elements in incident investigations that may make these groups suffer epistemic injustice.

## Methods

### Setting

Serious incident investigation practices in the Netherlands provide a fitting setting for our analysis, for the Dutch Health and Youth Care Inspectorate (HYCI) mandates healthcare organizations to engage all ‘knowledgeable actors’ of a serious incident in the subsequent investigation (Table 1) [31, 32]. As an effect, Dutch healthcare organizations—in particular Dutch hospitals—report high engagement levels of knowledgeable and involved staff members as well as patients and their relatives [9, 31].

**Table 1:** Explanation of Dutch national serious incident reporting system and definition of a serious incident

Internationally incident reporting systems and investigation activities vary. In the Netherlands, healthcare organizations are legally required to have internal incident monitoring systems in place and to report serious incidents to the Dutch Health and Youth Care Inspectorate (HYCI) within three days of discovery.
Dutch law defines a serious incident as an unintended and/or unexpected event related to the quality of care, having caused the death of, or serious harm to the patient. The European Commission upholds a similar definition [15]. Concrete examples include: surgery on the wrong individual or wrong body part; hemolytic transfusion reaction due to blood transfusion with major blood group incompatibilities; a fatal fall incident while receiving care or surgery.
These serious incidents are internally investigated by means of a Root Cause Analysis method, or similar form of investigation, in an attempt to learn from what went wrong. Healthcare organizations have eight weeks to investigate the event and send their incident investigation report to the HYCI. Upon request, the HYCI grants a six week extension if the eight week deadline is not feasible. In 2020 Dutch healthcare organizations reported 1680 serious incidents to the HYCI, 856 of these were reported by Dutch medical specialist organizations (80 hospitals, 700 private clinics, 20 rehabilitation centers and 14 abortion clinics). [12]
The legally binding serious incident investigation guideline dictates that all professionals involved in or with the incident should be involved in the investigation. Moreover, Dutch law obligates healthcare organizations to involve patients/family members in these incident investigations and disclose the findings. The HYCI oversees this process and monitors if all professionals/patients/families are indeed involved. The HYCI does not dictate what the involvement should look like but does stress the importance of involvement, normatively framing it as “a necessary ingredient to optimally learn from what has gone wrong” [11]. What’s more, involvement is framed as a way to facilitate healing and aid the process of reestablishing trust. [21, 31]

Between 2015 and 2018 the authors were engaged in a research program studying the incident investigation system of the HYCI. In this paper we draw from qualitative data gathered in five projects within this program, which examined different elements of Dutch incident investigation practices and HYCI’s supervision of these practices. Project details are summarized in Table 2. Combining the insights obtained from these different projects provided the authors a unique insight into incident investigation practices from the perspective of the actors responsible for organizing and executing serious incident investigations as well as assessing the epistemic value of a testimony: healthcare leaders, RCA investigators and HYCI employees. None of the data were collected with a specific focus on epistemic injustice but were focused either on getting a general understanding of the working of the system or were directed at more specific questions such as on the role of an independent chair of the committee. It was through abductively [44] analyzing the data that epistemic injustice came to the fore as a concept that helped in making sense of the data.

**Table 2:** overview collected data and analysis approach

Research project and fieldwork activities	Fieldwork activities conducted by (author initials)	→	Data analysis phase 1	Data analysis phase 2
*Project 1* (Feb–Aug 2015)			Insights obtained from the policy documents, interview transcripts and observation reports, were used to construct a detailed overview of serious incident investigation practices in the Netherlands. Special attention was paid to map out: - Social processes: actors involved, their tasks, responsibilities and interactions - Structures: formal and informal processes / organization of activities ( i.e., how are actors approached, how and where are interviews organized etc.), legal framework	Transcripts were coded deductively using a coding schedule, organized around the following themes: - What constitutes as ‘knowledge’ in incident investigations? - Who is thought to have knowledge? And why? / What are the “markers of credibility?” - How are testimonies collected, valued and processed? - Challenges / hurdles in the way, to embrace a testimony
33 h of observations incl. informal interviews at HYCI with inspectors responsible for overseeing incident investigation reports	JK			
Document analysis of protocols, guidelines and internal communication	JK			
*Project 2* (Apr 2015–Sept 2016)				
15 semi-structured interviews with diverse respondents (n = 18) in 13 Dutch hospitals. Respondents incl. healthcare professionals, incident investigators, quality managers	JK			
Document analysis of incident investigation protocols	JK			
*Project 3* (May–Nov 2016)				
31 semi-structured interviews with respondents from 4 Dutch elderly care and mental healthcare facilities, incl. organization leaders, quality managers, incident investigators and (external) incident investigation chairs (n = 24)	DdK, KG			
*Project 4* (Jan 2017–May 2018)				
8 semi-structured interviews with (former) HYCI inspectors involved in designing or monitoring the Dutch incident reporting system (n = 10)	DdK, KG			
Document analysis of HYCI policy documents on the development and aims of the incident reporting system	DdK, KG			
*Project 5* (Feb–Sept 2018)				
19 semi-structured interviews with diverse HYCI employees (n = 21), incl. inspectors, legal officers, program managers	JK, RB			
3 h of observations at the National Healthcare Report Center (HYCI incident report center)	JK			

### Study Approach and Analysis

We analyzed data collected in five qualitative studies of the Dutch incident reporting system. We draw from a total of 73 semi-structured interviews, 36 h of ethnographic observations at the HYCI and document analyses of policy documents collected at the HYCI and healthcare organizations. In all projects, respondents were purposely sampled [19]. Interviews were recorded with permission and transcribed verbatim. Observation field notes were transferred to observation reports and findings from the document analysis were processed into detailed written summaries.

**Fig. 1:**
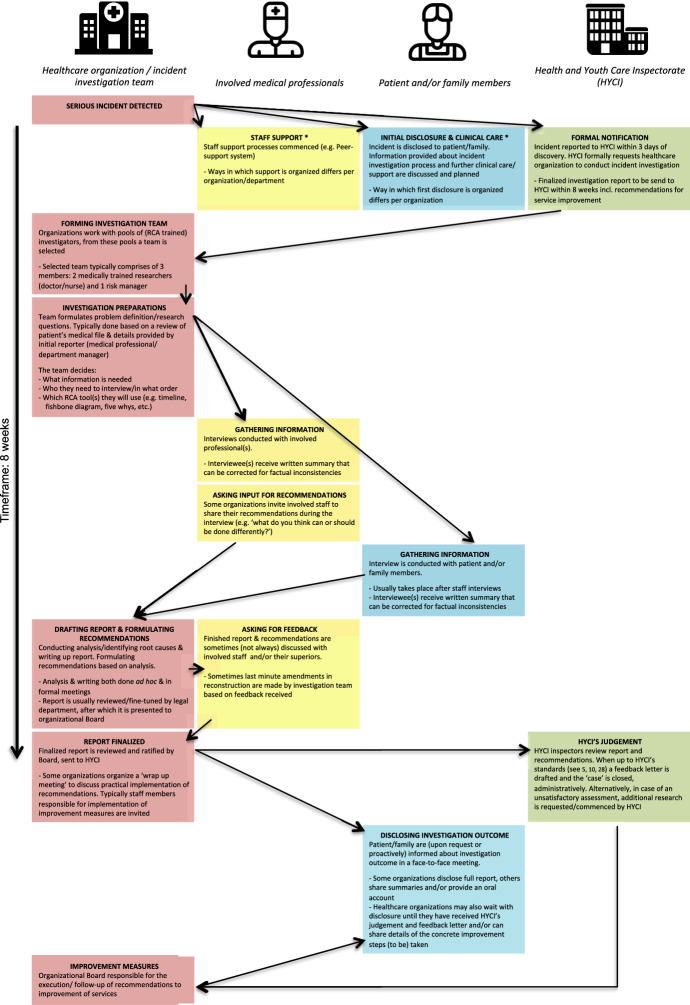
Overview of serious incident investigation practices in Dutch healthcare organizations *Although these stages are—strictly speaking—not part of the actual investigation process, they must be described in the final report. The HYCI monitors these steps. As a result, ‘staff support’ and ‘initial disclosure’ practices have become an integral part of serious incident investigation protocols in most Dutch healthcare organizations

Data analysis, conducted by JK and DdK, comprised of two phases (see Table 2). First, data were ordered and analyzed inductively (open coding) [22], to construct an overview of the structures and social processes involved with serious incident investigations, such as the sequential organization of all the investigation activities, who is involved and what are the tasks and responsibilities of the involved actors. These results were schematically mapped out in Fig. 1. Building on this overview, in the second phase, we specifically searched for examples of what constitutes as knowledge, who is thought to have relevant knowledge, who is actively heard, how testimonies are organized and how investigators value and interpret the different testimonies in the investigation process. Data were then coded deductively [19, 22] using themes that were informed by our review of the literature on epistemic injustice [2, 4, 5, 16, 17, 36, 40]. See Table 2 for an outline of these themes. In a ‘low technology approach’ [19], selected quotes and extracts were transferred into tables within Microsoft Word, ordering related findings. Lastly, the content in these tables was discussed with all authors to verify interpretations.

### Research Ethics

The type of research conducted in the overarching research program (anonymized observations, document analysis and retrospective interviews) are not subject to the Dutch Medical Research Involving Human Subjects act (Dutch abbreviation: WMO) [6]. For verification we refer to the guideline by the Central Committee on Research Involving Human Subjects as well as the waiver issued by the Erasmus MC Medical Ethics Committee (reference MEC-2018-054) [6]. All projects were performed in accordance with the Declaration of Helsinki research ethics principles. In all studies participants provided informed consent before the observations and interviews took place and were provided with the opportunity to review the transcripts. Data were anonymized.

## Results

The first part of the analysis allowed us to draw up a generalized overview of serious incident investigation practices in Dutch healthcare organizations, see Fig. 1. The overview displays the sequential order of activities that are geared into motion once a serious incident has been detected. Moreover, it shows which actors are involved, how they are involved and at what stage of the investigation process.

The second part of our analysis revealed that there are several structures in the incident investigation process that make professionals, patients and their families prone to suffer epistemic injustice. It is difficult to capture and translate these elements into rigid, clear-cut categories. In practice these structural elements overlap and, as we will show, permeate into each other. For the purpose of clarity, we use the sequential investigation order, as documented in Fig. 1, to present the elements we found.

### Investigation Preparations: The Gatekeepers Search for ‘Facts’

Shortly after a serious incident is detected, the organization where the incident has occurred, forms an investigation team. This team often consists of medical professionals (doctors/nurses) and risk officers/quality managers, whom have received training in RCA methodologies (see Fig. 1 and [31]). When deemed necessary, experts from outside of the organization are added to the team. Mandated by their organizational leaders, the team independently drafts a problem statement and decides: (1) what questions are central in the investigation, (2) what information they need to answer their question(s), (3) who they need to interview to retrieve this information, (4) in which order they wish to interview these people and (5) which RCA tool (e.g. timeline, fishbone diagram etc.) they will use to organize and interpret their findings. The independent status of the investigation team is reinforced by HYCI’s guidelines, which dictate that investigators should have no ties to the incident (see also [9, 32]). Document analysis of HYCI policy documents revealed that this ‘distance’ is associated with the ideals of securing impartiality.

It is specifically in the preparation phase of the investigation process that the epistemic privilege of the investigation team is clearly visible, for—by virtue of their mandated task—they act as gatekeepers and decide what questions and information are relevant. These individuals are held in esteem not because of their individual knowledge, but because of the group they now belong to: the investigation team. In that capacity they determine who holds relevant information, and who gets to speak up first. The team members, in other words, are the ‘interpreters’ of knowledge as they work towards shaping a narrative of the incident within the legal timeframe of 8 weeks.

The quest for root causes guides the investigators to look for and speak to all actors involved. In practice this translates to a search for ‘witnesses’; actors that have first-hand knowledge about the incident and/or the specific care process the incident relates to. Witnesses have to be reliable in the sense that they know what they talk about and can talk about it in meaningful ways. Other types of input or sources may be harvested but at the onset this input is seen as less valuable to the investigation. Examples are family members that are heard, to give them a chance to share their stories but *“they do not necessarily have a clear image of what has happened, what the facts are”* (Executive director elderly care facility, 06-10-2016) and they may not *“know how things go around here, what is normal”* (Quality & Safety manager mental care facility, 04-10-2016). Or, in another example regarding the input of professionals: when a senior doctor is heard instead of a junior doctor, because the first was perceived to be more experienced with the care process (Quality & Safety manager hospital, 12-07-2016). Here then, when there are multiple witnesses, investigators evidently consider some witnesses as more knowledgeable or credible—and thus reliable—than others.

Our interviews revealed that testimonies provided by actors that share verifiable facts to help determine the underlying root causes of the incident, are considered to be most knowledgeable. The emotions that interviewed actors may have, are frequently framed as problematic for a team’s fact-finding quest.

R: Sometimes they [patients/family members] are angry. Mad at everyone. Or they are dealing with a complex grieving process. Emotions like ‘they let my father die’. And then it becomes very difficult for us [as investigation team].

I: Can you tell me a bit more about that?

R: (…) Yes, well for example we had this case. A patient. He was filled with rage and grief. One minute he was here, the other he was there [pointing at different places in the room], literally, like in the room but also in his story. Just all over the place. (…) I mean it was terrible, ter-r-ible what happened to him, but he, eh, was also very stuck in these emotions. He had a [negative] opinion about everything.

I: Was his testimony useful?

R: Well it was, because he had a chance to share his story. (Quality & Safety manager hospital, 20-09-2016)

Here listening to the patient is seen as useful to allow him to voice his experience but he is not judged to be a knowledgeable participant in the collective practice of interpreting and understanding what went wrong [5]. Him being ‘all over the place’, angry and in grief makes his story not only less intelligible but also less credible—emotions create distance from, or possibly distort, the facts. The quote sheds light on the way emotional patient and family testimonies are assessed. Providing space to let the emotions out is seen as useful, for purposes of healing and regaining trust (see also [31]), but being emotional might mean being perceived as less reliable, risking one’s testimony to be devalued.

For professionals the same ‘logic’ applies. Our respondents noted that it is understandable if professionals feel sad, nervous and/or anxious, but these emotions must be contained as much as possible. *“You don’t want those emotions in the investigation”* (Quality & Safety manager hospital, 12-07-2016). Emotions are thought to cloud someone’s vision, contaminating their testimony. These findings resonate with other studies on epistemic injustice that report how emotional testimonies are often discredited and perceived to have little or less epistemic value. Moreover, claiming someone is too emotional is a common way—at times even a deliberate strategy—to downplay the value of someone’s input [4, 5, 16].

### Gathering Information: Providing the Right Format?

As Peerally et al. have noted, a key problem surrounding RCA investigations is that the information obtained from the interviewed actors is influenced by their willingness and ability to provide relevant data as well as the nature of the relationships between interviewees and investigators [41]. Aside from this being a challenge in itself to attain ‘high quality RCA investigations’ [41], our analysis revealed that the fact that the investigators decide what is relevant and dictate the format in which information should be provided and collected, are obstacles to achieving epistemic justice for professionals, patients and their families. With respect to format, four different points came to the fore.

First, the predefined eight-week timeframe in which investigation teams are to conduct their investigation and produce a final report, can give rise to epistemic exclusion. Respondents mentioned that most patients and family members are willing to contribute to the investigation process, but in practice some are not ready to provide their testimony within eight weeks after the event because they are still processing the emotional impact of the incident. As a result, these testimonies are excluded entirely. Moreover, when we recognize that framing someone as ‘too emotional’ can be a deliberate strategy to discredit someone’s epistemic contributions [4, 5, 16], actors run the risk of being excluded or undervalued if other parties, in this case the investigation team, assesses such ‘emotional readiness’.

Second, the composition of the incident investigation team sitting across an interviewee, influences the way in which that interviewee is at ease, how a story is shared and what is shared. Our respondents noted that nurses and junior doctors were often visibly more nervous than interviewed doctors *“who sit there talking to their peers, so there is less pressure”* (Medical doctor/RCA investigator hospital, 18–08-2016). A professional recalled:

It was really daunting [to be interviewed]. Just because I knew that I would be sitting across our medical director [one of the investigators]. (Personal Care attendant mental care facility, 12-10-2016)

Quality and Safety managers and organizational leaders stressed that incident investigators are trained to conduct interviews in an open and non-judgmental manner. What’s more, these respondents explained that, especially in the case of complex incidents, the investigation teams are strengthened with an extra member and/or a ‘very experienced investigator’ to ensure information is collected according to high standards. In practice these experienced investigators are often in positions of authority (see also [8]). As the earlier quote reveals, this can make interviewees nervous and careful in what words to use. A ‘daunting’ setting where actors feel affected by interpersonal power-imbalances might inhibit someone to clearly express him- or herself.

Also, knowing that the final report will be sent to the HYCI can make some professionals wary about giving information:

I know for a fact that if I ask a nurse ‘do you feel any barriers to phone the on-call doctor during a night shift?’ that there will be nurses that say: ‘yes, there are doctors that are difficult to approach.’ (…) Once we start an incident investigation and I ask that same question, there is not a single nurse that’s going to tell me that these difficulties exist. I’m just not going to get that on record, because this is an investigation. The report will go to the Inspectorate. You’re [the nurses] not going to say that! (Quality & Safety manager hospital, 10-08-2015)

These relationships and power dynamics can influence the way testimonies are given and can render particular actors prone to suffer epistemic injustice, e.g. when actors self-censure their testimonies because they do not feel safe enough to share openly. To be clear, the relational setting does not automatically trigger epistemic injustice, but relational power dynamics have the potential to impact some groups in particular ( i.e., nurses or junior doctors), hampering their ability to articulate their stories. On top of that, actors’ testimonies might be unjustly discredited given their position (as being ‘just’ juniors or nurses) or when their nervousness is interpreted as uncertainty or doubt.

A third point with regard to format is the nature in which the ‘collection of information’ is shaped. The investigators have usually prepared an extensive list of (closed) questions before the interview commences; questions in line with their quest for root causes. Such a strict interview guide may not provide the appropriate amount of space for an actor to share all he or she wants to share:

I had the feeling I couldn’t really say what I wanted to say. (…) They [investigators] did explain that the goal was to learn, they explained that well. And it wasn’t their tone or anything, but the way we kept going over the same points, going back to their specific questions. While I had the feeling that I had already answered their questions and all my other points, the things I contributed, didn’t receive any attention. When we were finished, I was like ‘this wasn’t a pleasant conversation’. That was just my experience, even though they were really friendly. (Personal Care attendant mental care facility, 12-10-2016)

For patients and family members similar experiences are likely. Most teams, we learned, decide to speak to the patient and family after the team has drafted their first RCA timeline or fishbone diagram. A draft based on the input from involved professionals and a review of medical file(s). Inviting patients and families to ‘confirm’ elements on the predefined timeline and/or only inviting testimony on specific parts of the care process, may not do justice to the complexity and richness of their stories. Moreover, respondents explained that investigators often have to manage patient and family expectations. When they share experiences and concerns about issues—which investigators interpret as ‘side-issues’ that are not part of the team’s investigation focus—investigators struggle to take such testimonies into account.

The fourth point on format relates to the setting in which the interview takes place, for this too can influence an actor’s (emotional) ability to share what he or she wants to share. Interestingly, our analysis revealed that patients and families are often asked where the interview can best take place; at home or the healthcare facility. In comparison, professionals are not offered an option. A nurse shared her experience of when she was asked to re-enact her actions with a colleague, in the same room the incident had transpired. The investigators watched, took notes and ran through their pre-formulated questions:

At that moment, the questions they asked, I felt it was so inconsiderate. The investigators didn’t realize how intense this confrontation was. They were just trying to solve a ‘thing’, but I don’t think they were aware what the impact of their approach was. (…) In your mind you’ve gone over it, over and over; how could this have happened? And then you stand there in that room, and they bring in the stretcher [the ‘prop’ needed to show the investigators what had happened]. We couldn’t hold back our tears. (Nurse elderly care facility, 13-10-2016)

The quote reveals that in the interviews, investigators create or search for spaces where causes and ‘facts’ can be observed and documented. The emotions that are triggered by the same place or setting, as we have shown earlier, can influence the way in which a testimony is valued by investigators.

### (Not) Asking Input for Recommendations

When incident investigators ‘gather information’ to construct their narrative of the event, the interviews with involved professionals are sometimes embraced as a moment to ask input for recommendations towards service improvements. Some healthcare organizations have made this an integral part of the incident investigation process; stimulating professionals to ‘speak up’ and ‘think along’. There are, however, also organizations that avoid asking input for recommendations, stressing the importance of the investigation team’s independence.

I: Who formulates the recommendations?

R: The investigation team.

I: Ok. And do you ask for input from the actors involved?

R: No. No. Noo. We are independent investigators. So, as a team you really have to do that yourself. (Medical doctor, incident investigator hospital, 18-08-2019)

The importance of having—or maintaining—independence also plays a role in organizations that do encourage voice:

We do ask for input, but if that input is used is another matter that’s up to the investigators. (Quality & Safety manager hospital, 28-06-2016)

In this phase of the investigation process, independence was not only thought of as an imperative for validity and reliability. Rather, the team’s task to formulate SMART (Specific, Measurable, Attainable, Related and Timely) recommendations in line with their defined root causes—one of HYCI official requirements (see also [9, 32])—is often interpreted by them as stressing the team’s autonomy. As an effect, the regulatory requirements make it difficult for the team to assign equal value and/or ‘really listen’ to suggestions for recommendations. Moreover, even when input is sought, treating proposed ideas for improvement as mere input that needs to be weighted by the investigation team, this one-way approach of gathering and weighing/interpreting data from involved actors does not stimulate a process of shared learning and improving.

Ultimately then, in both scenarios, the investigation team’s epistemic privilege is underscored, which inhibits a shared practice of learning. In practice, as Iedema et al. have shown, this can cause a disjunction between recommendations and their workability and implementability at the sharp end [27]. But, aside such practical implications, entirely excluding or ignoring parts of someone’s ideas for improvement(s) are suggestive of epistemic injustice.

### (Not) Asking for Feedback and Disclosing Investigation Outcome

Our analysis revealed that organizations struggle with the ways in which feedback loops and dialogues between investigators and involved actors can be organized and processed in the last phase of the investigation routine. Interviewed professionals are typically asked to review the final version of the report and subsequent recommendations, but like the feedback requested on the earlier interview summaries in the ‘gathering information’ phase, their feedback should—ideally—be geared towards rectifying factual inconsistencies. It is common, however, that professionals do not agree with (parts of) the finalized narrative and/or feel their testimonies were misunderstood, not taken seriously or ignored entirely.

I thought the report was one sided, to be honest. Because the points I had made, about broader policy issues and stuff like that, those were not in the report. (Personal Care attendant mental care facility, 12-10-2016)

Professionals who disagree with the conclusions are sometimes described as *“not being that far along with patient safety thinking”* (Quality & Safety manager hospital, 29-06-2016). Or, in another telling example, one healthcare organization purposely did not invite the interviewed professionals to the ‘wrap up meeting’ to discuss the final report because *“they are too emotional; (…) doctors are just not used to hearing, in the presence of lay people, that something hasn’t gone right”* (Assistant manager Quality & Safety hospital, 20-09-2016). A narrow feedback loop or lack of dialogue then can cause or amplify feelings of frustration and injustice.

Patients and family members are usually not asked for feedback on the final report. Respondents note that this is partly due to time constraints, but they also argue that they fear patients and/or families will feel the investigation is corrupt or a hoax if preliminary conclusions are changed in the final stage of the investigation. Dutch healthcare organizations do increasingly disclose the investigation reports to patients and families (redacted or not), once it has been sent to the HYCI [9]. Our interviews show, however, that patients and families do not always agree with the conclusions of the report. Voicing these concerns can cause investigators to label them as ‘difficult’, ‘unintelligent’ and/or ‘looking for someone to blame’.

The stereotypical labels attached to actors voicing concerns or actors with a dominant take on what has happened can deflate their input earlier on in the investigation process. The illustrative anecdote below reveals this risk: a mother carrying the label ‘difficult parent’ felt her testimony was not taken seriously and protested once the finalized report was disclosed to her:

She [the patient’s mother) was a ‘difficult’ parent, like you just come across in healthcare sometimes. Perceived as difficult in the sense that she had her own vision and I don’t know what else. (…) [T]he parent objected to our conclusions (…) We concluded that the anesthesiologist had “acted professionally” during a cardiopulmonary resuscitation, [but] the parent disagreed. Listening to her, I thought “You’re right”. The anesthesiologist had acted professionally in her medical expertise, but she had not communicated professionally with the patient’s representative. So, I get why our assessment of “acting professionally” upset [that parent]. I said, “You’re right, ma’am.” (External Chair investigation committee mental care facility, 13-10-2016)

This quote hints at the potential value for investigators to ask for and listen to feedback, even if the experience that is shared (or objection made) does not specifically relate to ‘hard’, verifiable facts. The ‘difficult parent’ label had prevented the investigators from taking the mother’s earlier voiced experiences seriously. In epistemic exchanges, continued dialogue can help actors to further their understanding and facilitate learning [2]. Moreover, it can help to do justice to the epistemic contributions that have been shared.

## Discussion

Earlier studies have shown that the HYCI has successfully stimulated healthcare organizations to collect input from all knowledgeable actors in their serious incident investigations; professionals, patients and their families [9]. Our analysis shows however that providing testimony does not always mean that this testimony is heard, understood or valued. What’s more, who is recognized as a knowledgeable actor and seen as holding relevant information for the incident investigation is not a ‘given’. Rather, this is determined by incident investigators and the institutionalized structures in which these investigators work. We identified several structures that can promote or hinder an individual participant’s knowledge contribution in the process of an incident investigation. First, the RCA tools used in incident investigations steer investigators to map out timelines and fishbone diagrams to work towards a linear narrative of the incident. The construction of a linear narrative makes investigators prone to displace or disregard different interpretations to how events have unfolded and have been experienced [38, 41]. Moreover, the RCA tools used, drive investigators to search for verifiable facts or other forms of ‘hard’ evidence. As a consequence, actors that provide testimony outside the scope of such a timeline and/or share experiences that are not verifiable, risk being seen as less relevant. This can unjustly disqualify testimonies that could have harvested information regarding latent organizational factors that contributed to the adverse event occurring when it did. Also, the format used to collect testimony, i.e., only asking specific questions, the setting in which the interview takes place, the relationships between the actors involved, may not suit the kind of testimony an actor wishes to share and pose a barrier for a speaker to articulate their story clearly [5]. The incident investigators formal task to formulate recommendations towards service improvements in line with the identified root causes,—a regulatory requirement—can inhibit the team to use ideas for service improvement(s) provided by professionals. Lastly, investigators also carry personal and structurally induced biases towards professionals, patients and families, judging them to be ‘too emotional’, ‘unintelligent’ or ‘not committed to patient safety’. These biases influence how investigators assess and value an actor’s testimony. The process of assessing and valuing testimonies by an independent investigation team can transform the participation of professionals, patients and families into a one-way process, instead of a social process of shared learning from incidents. Taken together it is evident that, even though the HYCI has provided an—arguably successful—impulse to multi-voiced engagement in incident investigations, in practice institutionalized structures at the local and national (regulatory) level, pose barriers to do justice to and facilitate shared learning from all testimonies.

Epistemic injustice in incident investigations, then, can be triggered through the prejudice of incident investigators but also by the way in which an incident investigation is structured, hindering testimonies from being articulated or heard clearly, or not at all ( i.e., epistemic exclusion). The structures we have presented in the analysis, are mostly likely to trigger testimonial forms of injustice. That is, specific groups of actors may suffer from a credibility deficit due to the stereotypes attributed to them during the different stages of the incident investigation process, i.e., a junior doctor is less reliable than a senior doctor, or testimonies are too emotional etc. Whilst it is legitimate to value an experienced person’s knowledge higher than a novice, this should be based on the knowledge presented. Epistemic injustice occurs when an individual’s knowledge is judged through the lens of prejudice: the junior doctor’s input is judged less reliable because she is junior, not because of what she says. Hermeneutical injustice may also rise but our study does not allow us to come to that conclusion for we do not know if testimonies are deflated as a result of lacking (conceptual) resources although the quote about the patient ‘being all over the place’ does suggest this in the sense that informational and conceptual clarity was asked for. Nonetheless, in whatever form, the identified structures can be problematic as epistemic injustice can hinder learning from serious incidents and prevent doing justice to the lived experiences and ideas from healthcare professionals, patients and their families. In a time when healthcare organizations are seen to have a duty to learn about what happened from multiple perspectives [13], and there are popular calls to ‘better involve patients’ [14, 28], to ‘take patients seriously’ [39], and ‘value everyone’s language equally’ [43], an important contribution of this paper is to illustrate the institutionalized structures that can complicate such efforts. Calling for practices of individual testimonial and hermeneutical justice is understandable, but such calls have to be accompanied by an awareness for the structural conditions that allow for such practices [2, 36].

Epistemic injustice is problematic beyond the scope of learning from incident investigations, for—as Fricker explains—if someone has the experience of not being taken seriously as a source of information, they can lose their confidence in their ability to obtain and transmit knowledge [17, 24]. They may silence their own voice or undermine their own experience, which can be detrimental for efforts to further patient safety and organizational safety cultures more generally, as both rely on open communication. Moreover, not including the knowledge of patients or lower status professionals can reproduce existing inequalities.

Tackling epistemic injustice is challenging, but being familiar with the concept itself can be a first step to understand what is required in practice to operate in a way that works against it [17]. In any given situation, being mindful of our own prejudice and how this influences our ability to understand and (under)value what someone is saying, is important. Initiating policies to train incident investigators, to stimulate such reflective thinking and challenge their biases can be helpful. Such schooling, for instance in the form of role-play, should be offered on top of investigators’ formal RCA training. Such training should include talking to respondents that are emotional or reluctant to share information because of status differences. Clearly though, (enhancing) personal reflectivity and/or genuine willingness to do justice to someone’s testimony is not enough. For, as we have shown, there are social and institutional structures that promote biases, cause epistemic exclusion and prevent credible actors from being valued as such.

In light of our analysis, we have three recommendations for healthcare regulators, policy makers and incident investigation practices. First, healthcare managers can map out the organizational structures that can make actors prone to suffer epistemic injustice. In line with Carel and Kidd, recognizing structural epistemic asymmetries—between doctors and nurses, doctors and patients, investigators and interviewed actors, etc.—is crucial when one wishes to equally solicit their contributions within epistemic practices like incident investigations [5]. Critically appraising the practical elements in these structures, such as the composition of the incident investigation team, the format in which actors are to voice their experiences, and how these formats can possibly be more accommodating, may be a good first step. For example, it could help if the involved healthcare professionals are interviewed by their peers. So, a junior doctor by a junior doctor, instead of by senior doctor to whom the junior might not disclose their experiences in the same manner or who’s testimony might be undervalued because of prejudice towards junior doctors. Also, at the start of an investigation, investigators may want to seek input for the research questions with all those directly concerned, to prevent an all too narrow framing of the problem statement. Any controversies or conflicting experiences voiced in the interviews can be captured in the report rather than edited out; in this way, justice can be done to the different experiences and perceptions of involved actors. Second, building on the first recommendation and Dekker’s notion of ‘just culture’ [7] it is crucial to ask the actors involved: what do you need to be able to share your testimony? Instead of simply inviting actors to tell their story, or only asking for specific details of their story. For example, ‘replaying’ an incident can be helpful but investigators should recognize that this puts emotional burden on the professionals involved. Assisting them in this and maybe also interviewing them on another occasion might be helpful in preventing their testimony to be discredited. Investigations can then also be a part of the recovery process instead of only a process of fact-finding. Third, encourage and organize continued dialogue and feedback loops, for learning from mistakes is an on-going social practice [9, 33]. For example, we found that the timeframe of the RCA poses barriers to continued epistemic exchanges. This is something that could be negotiated with the external regulator. Also, the practice of having the committee set the epistemic boundaries of the RCA— i.e., by posing the questions that need answering—instead of involving professionals and patients concerned in this creates tensions for including their testimonies later on in the process. This can be easily remedied by having a more inclusive process at the start as well as the wrap-up of the investigation. Even with such changes, epistemic injustices of course might still occur; as Maitra has noted, giving full credit to all possible perspectives is too much to ask [34]. Moreover, pressure from outside, for example the media, might prevent full disclosure of different perspectives. Nevertheless, the proposed changes might make both investigators and supervisors more aware of possible epistemic injustices.

In our quest to understand how testimonies are collected and knowledge is appraised in incident investigations, we have focused the main part of our analysis on ‘the hearer’s point of view’, i.e., incident investigators, managers and healthcare leaders. We have a limited understanding of experiences of epistemic injustice of people giving testimony. This is an important limitation of our study. More research should be done to explore and understand how patients, families and involved professionals experience epistemic injustice as such insights can help strengthen efforts to address these issues. Also, comparative research can be useful to further our understanding of the structural mechanisms that trigger epistemic injustice in different healthcare settings, including their incident investigation systems. As incident investigation systems have been introduced all over the world to facilitate shared learning [14, 32] and foster healing [7, 10, 31, 47] such an analysis is called for.

## Conclusion

In this article we have shown that inviting someone—whether a healthcare professional, patient or family member—to share their experience about healthcare encounters or service delivery, does not automatically guarantee that this testimony is understood or valued by the hearer(s). Institutional structures can prevent someone’s relevant knowledge from being recognized as such. As we have rendered epistemic injustice in incident investigations tangible, we hope our analysis does not discourage. Rather, we encourage healthcare providers, policy makers and regulators to use these insights to further their commitments to multi-voiced engagement in healthcare. This can improve learning from incident investigations and—more broadly—facilitate quality and safety improvements that do justice to the experiences of healthcare professionals, patients and their families.
